# Paraly: An (annotated) dataset for exploring the concept of paralysis (fr. ‘paralysie’) in a digital corpus of French Literature

**DOI:** 10.1016/j.dib.2025.111577

**Published:** 2025-04-26

**Authors:** Daniela Kuschel, Renat Shigapov

**Affiliations:** a University of Mannheim, Romanisches Seminar, Literatur- und Medienwissenschaft, 68131 Mannheim, Germany; b University of Mannheim, Forschungsdatenzentrum, Universitätsbibliothek, 68131 Mannheim, Germany

**Keywords:** French Literature, Literary studies, Cultural studies, Discourse analysis, Metaphorology, Narratives of Im/mobility

## Abstract

The dataset consists of three corpora (full texts and metadata) of French Literature of the 18th, 19th and 20th century containing figurative and concrete linguistic references (annotations) to the concept of paralysis. The texts originate from the collection “Les classiques de la littérature” [The Classics of Literature] maintained on Gallica, the public digital library of the National Library of France (BnF). The dataset contains original OCR-ed texts with their metadata, the annotations of text excerpts containing the character sequence ``paraly”, the annotation guidelines, a model and application for automatic annotations, and codes for data collection, extraction, processing, and training. After the data collection from Gallica, we used the open-source software *CorpusExplorer* to automatically annotate part-of-speech and lemma information for each text and to create a separate text corpus for each century. The *CorpusExplorer’s* ``Key Word in Context'' (KWIC) feature was used to search for the character sequence “paraly” within each text/corpus. Based on the results, new century-specific corpora were created, containing only texts with this characteristic. With the help of *OpenRefine* we cleaned the tables with the metadata to ensure consistency of the entries. The text passages containing the set of characters “paraly” have been manually annotated. A multilabel classifier was then trained using the annotated data and the flair-library, and an application with a graphical user interface was deployed at Hugging Face. The provided dataset facilitates a better understanding of the associated research project on the French literary and cultural history of paralysis. The methodological approach can be adapted to generate new research datasets tailored to other studies.

Specifications Table

**Table:** 

Subject	Humanities (General)
Specific subject area	French Literary and Cultural Studies.
Type of data	Table, Code, Text Corpora. Filtered, Processed, Annotated.
Data collection	The webpages with books from the collection “Les classiques de la littérature” at Gallica, described in the next section “Data source location”, were scraped to get data about authors and links to their pages. Then, we used the SRU-interface https://gallica.bnf.fr/SRU to get the metadata of all the books in the collections. Finally, we downloaded 13,524 books using https://gallica.bnf.fr/ark. The whole process is documented in the Jupyter Notebook get_OCRed_books_from_gallica.ipynb provided in this dataset. Some works of the collection can only be accessed on the library site. Therefore, errors occurred in the download process due to copyright restrictions of the BnF. Accordingly, these texts were excluded.
Data source location	Gallica, digital library of the Bibliothèque National de France (Paris, France): https://gallica.bnf.fr/html/und/litteratures/les-classiques-de-la-litterature-acces-par-periode?mode=desktop • 18th century: https://gallica.bnf.fr/html/und/litteratures/les-classiques-de-la-litterature-du-xviiie-siecle • 19th century: https://gallica.bnf.fr/html/und/litteratures/les-classiques-de-la-litterature-du-xixe-siecle • 20th century: https://gallica.bnf.fr/html/und/litteratures/les-classiques-de-la-litterature-du-xxe-siecle
Data accessibility	Repository name: MADATA Data identification number: https://doi.org/10.7801/471. Direct URL to data: https://madata.bib.uni-mannheim.de/471 and https://github.com/UB-Mannheim/paraly.

## 1 Value of the Data

• The dataset ensures that the associated research project is reproducible.
• Researchers can reuse the data for other research on French texts that relate to paralysis in different ways. The digital corpora with full texts and metadata can be used as a basis for linguistic questions, metadata-related interests, and historical and cultural studies.
• The annotated text passages and models can be especially useful for the investigation of metaphorical concepts but also for gaining more insights on how physical paralysis is addressed in (literary) texts. The related metadata can provide useful contextual information (author, editor, year, text/library classification, etc.) for this purpose.
• The methodical procedure can be reproduced with the help of the descriptions and codes, which allows other researchers to create their own text corpora and investigate on similar research questions.

## 2 Background

The dataset [1] was created in relation to a study on the French literary and cultural history of paralysis. The challenge at the beginning of the project was to identify texts in which paralyzed fictional characters appear and, more globally, all kinds of textual representations of paralysis. Without a large corpus of full texts that could be browsed digitally, this endeavor would not have been possible. The text corpora for each century of interest to the study form the basis for the study questions and permit evidence-based answers. As the collection “Les classiques de la littérature” [2] is a living collection that is maintained and expanded on a regular basis, the dataset is based on a snapshot (reference date: 24.11.2022).

Amongst the various research questions one is of major interest: the difference between concrete and figurative references to the concept of paralysis, necessary for its theorization. That is why annotating this information was indispensable.

## 3 Data Description

The dataset follows a structured hierarchical organization. Each folder is named according to its function, and subdirectories correspond to specific tasks, such as annotation, training, evaluation, and (meta)data collection. File names use a consistent format. We use prefixes to indicate the century of the corpus (e.g., 18_, 19_, 20_). The paraly-dataset has the following structure: paraly/ is a root folder

├—— docs/ –> a folder with documentation
│ └—— paraly_annotation_guidelines.pdf –> the guidelines for annotation
├—— data/ –> a folder with data
│ ├—— training/ –> a folder with training data
│ │ ├—— train_fasttext_dataset.txt –> training subset in FastText format
│ │ ├—— test_fasttext_dataset.txt –> test subset in FastText format
│ │ └—— dev_fasttext_dataset.txt –> dev subset in FastText format
│ ├—— model/ –> a folder with evaluation, training logs, and a model
│ │ ├—— training.log –> a training log-file
│ │ ├—— test.tsv –> an evaluation on test subset of data
│ │ ├—— paraly_camembert_large_multilabel.pt –> a classification model
│ │ ├—— loss.tsv –> values of loss function and metrics during training
│ │ └—— dev.tsv –> an evaluation on dev subset of data
│ ├—— errors/ –> a folder with errors
│ │ └—— all_metadata_errors.csv –> metadata of files, which were not collected
│ ├—— corpus/ –> a folder with corpus
│ │ ├—— 20_paraly_metadata.tsv –> a TSV-file with metadata for 20th century
│ │ ├—— 20_paraly_data_TEI.xml/ –> a folder with corpus for 20th century in TEI format
│ │ ├—— 20_paraly_corpus.cec6 –> corpus for 20th century in cec6 format
│ │ ├—— 19_paraly_metadata.tsv –> a TSV-file with metadata for 19th century
│ │ ├—— 19_paraly_data_TEI.xml/ –> a folder with corpus for 19th century in TEI format
│ │ ├—— 19_paraly_corpus.cec6 –> corpus for 19th century in cec6 format
│ │ ├—— 18_paraly_metadata.tsv –> a TSV-file with metadata for 18th century
│ │ ├—— 18_paraly_data_TEI.xml/ –> a folder with corpus for 18th century in TEI format
│ │ └—— 18_paraly_corpus.cec6 –> corpus for 18th century in cec6 format
│ └—— annotations/ –> a folder with annotations
│ ├—— 20_paraly_annotations_v1.xlsx –> a XLSX-file with annotations for 20th century
│ ├—— 20_paraly_annotations_v1.csv –> a CSV-file with annotations for 20th century
│ ├—— 19_paraly_annotations_v1.xlsx –> a XLS-file with annotations for 19th century
│ ├—— 19_paraly_annotations_v1.csv –> a CSV-file with annotations for 19th century
│ ├—— 18_paraly_annotations_v1.xlsx –> a XLSX-file with annotations for 19th century
│ └—— 18_paraly_annotations_v1.csv –> a CSV-file with annotations for 18th century
├—— code/ –> a folder with codes
│ ├—— training/ –> a folder with a training code
│ │ ├—— train_fc.py –> a training code using flair-library
│ │ └—— README_training.md –> a README-file for training code
│ ├—— splitting/ –> a folder with a code for splitting the data
│ │ ├—— prepare_training_data.py –> a code for splitting the data
│ │ └—— README_splitting.md –> a README-file for splitting the data
│ ├—— merging/ –> a folder with a code for merging data
│ │ ├—— Merge.ipynb –> a Jupyter Notebook for merging data
│ │ └—— README_merging.md –> a README-file for merging data
│ ├—— extraction/ –> a folder with scripts for data extraction
│ │ ├—— query.txt –> a file for configuring search spans and queries
│ │ ├—— Starten.bat –> an execute script for query extraction
│ │ ├—— Skript.cecs –> a script for CorpusExplorer
│ │ └—— README_extraction.md
│ └—— collection/ –> a folder with scripts for data collection
│ ├—— get_metadata_for_corpus.ipynb –> a notebook for collecting metadata for corpus
│ ├—— get_metadata_for_all_books.ipynb –> a notebooks for collecting metadata for all books
│ ├—— get_OCRed_books_from_gallica.ipynb –> a notebook for downloading books from Gallica
│ └—— comment_metadata_in_html_files.ipynb –> a notebook for processing the metadata
│ ├—— app/ –> a folder an application with graphical user interface
│ │ ├—— app.py –> a python file with a gradio-application
├—— README.md –> a README-file for the whole project
└—— LICENSE –> a file, which specify licenses for all parts of this root folder

We used several common formats to ensure interoperability and reproducibility. The corpus is stored in the Text Encoding Initiative (TEI) format, a widely used standard for encoding textual data. Metadata and annotations are provided in structured tabular formats (TSV/CSV/XLSX). Training and test data for machine learning models are stored in the FastText-compatible format, facilitating direct use with text classification frameworks.

The dataset includes manual annotations stored in .xlsx and .csv files. Labels are assigned according to predefined annotation guidelines (see paraly_annotation_guidelines.pdf). The annotations are categorized into three label types: “c” (concrete), “f” (figurative), and “cf” as an inter-category.

Table 1 summarizes the label distribution across different centuries.

**Table 1:** Counts of labels in paraly-corpora in every century.

Century	Count of “c”-labels	Count of “f”-labels	Count of “cf”-labels	Count of all labels
18th	60	52	43	155
19th	508	929	158	1,595
20th	37	99	23	159

The corpus spans from the 18th to the 20th century, containing a total of 1,152 TEI-encoded files, with 99 files from the 18th century, 961 files from the 19th century, and 92 files from the 20th century.

## 4 Experimental Design, Materials and Methods

### 4.1 Data and metadata collection

The collection on Gallica is organized by author and/or period. The webpages with books from the collection “Les classiques de la littérature” at Gallica were scraped to get data about authors and links to their pages. Then, we used the SRU-interface https://gallica.bnf.fr/SRU to get the metadata of all books in the collections. Finally, we downloaded 13,524 books using https://gallica.bnf.fr/ark. At the end, we got all OCR-ed books of the three centuries of interest (18th, 19th and 20th century) as html files. The whole process is documented in the Jupyter Notebooks get_OCRed_books_from_gallica.ipynb, get_metadata_for_corpus.ipynb, and get_metadata_for_all_books.ipynb.

### 4.2 Data filtering

The html files per century were imported into the open-source software *CorpusExplorer* [3] and part-of-speech and lemma information was automatically annotated using the option “tree tagger without phrases” for the language “French”. The aim was to create three separate corpora within the software to facilitate the search for the set of characters we were interested in.

In this step, the problem appeared that the metadata was part of the texts itself, presented as a first page in each document. Therefore, the *CorpusExplorer* could not recognize it as metadata.

The problem did not influence the search for the set of characters “paraly”, so we decided to continue the filtering process and add the metadata later. At any time, the texts could be identified via the ark-identifier provided by the BnF. The *CorpusExplorer* assigns GUIDs to all texts, too, so that unique identification was never a problem.

We used the tool “frequency analysis” of the *CorpusExplorer* to realize the search for the set of characters because the KWIC tool of the software only supports the search for full and exact words. This approach allows us to include words with spelling mistakes, even if the errors do not affect the specific set of characters itself. Spelling or OCR recognition mistakes in the set of characters “paraly” were tested manually for the most frequent errors, e. g. the mix-up of “f” and “l” due to the typefaces of older French texts.

Using the ‘Snapshots’ function, we saved the results and created a corpus for each century containing only those texts with the set of characters “paraly”.

### 4.3 Metadata merging

During data and metadata collection, we obtained a list with all metadata for each century. Out of this list, we filtered the metadata for the paraly-corpora.

Using the function ‘Korpusverteilung: Bearbeiten’ [‘distribution of corpus: edit’] of the *CorpusExplorer,* we exported TSV files for each corpus containing the ark-identifier and the GUID of each text - this was the only metadata available during the import process (see “Data filtering”).

These lists have been compared to the list with all metadata and merged via the inner-merge function of the python library “pandas”. As a result, we got three lists of metadata, one for each century and corpus.

### 4.4 Data cleaning

We cleaned the entries of the three metadata lists with *OpenRefine* [4] and added a short title (‘last name_First three to five words of title’), which allows us to rapidly identify a text during analysis. The completed and cleaned tables in the TSV format (“18_paraly_metadata”, “19_paraly_metadata”, and “20_paraly_metadata_”) were then reimported into the *CorpusExplorer*, (via ‘Korpusverteilung: Bearbeiten‘) and joined with the texts. It then became possible to export the corpora with metadata for each century.

It was essential to verify once more whether these corpora contained duplicates, meaning identical text passages appearing in different texts. This can occur, for example, if the corpus includes different editions of the same text or if a text is published both individually and as part of an author’s collected works. Our approach was as follows: For each author, we displayed the text passages containing “paraly” in the *CorpusExplorer* and compared them. We retained the oldest version or edition of a text and removed any duplicates.

Finally we created and exported the cleaned final corpora: “18_paraly”, “19_paraly”, and “20_paraly”. Given that the software *CorpusExplore*r is not widely used, we decided to provide the final corpora in the TEI-XML format additionally, ensuring broader accessibility and usability.

### 4.5 Data annotation

For the annotation process it was first necessary to get the text passages that contain “paraly”. To keep the process simple, we decided to use excel as an annotation tool. With the help of the script skript.cecs we could automatically retrieve the text passages from the corpora. We decided to use three categories: “c” (concrete), “f” (figurative), and “cf” as an inter-category.

The following annotation guidelines were used:

• “c”: refers to concrete instances of physical paralysis due to illness or accident, permanent or temporary, regardless of the specific type.
• “f”: refers to a purely figurative sense, e.g. when resources, actions, feelings, ... are paralyzed.
• “cf”: is a hybrid category used for cases where there is an explicit reference to a concrete manifestation of paralysis that is used metaphorically, e.g. a body part like the tongue is described as paralyzed but does refer figuratively to the inability to speak. Often “cf” refers to Bible stories or art objects (e.g. paintings, sculptures, ...) where paralyzed persons are represented. But it can also describe temporary physical paralysis caused by medication, electric shocks or magic.

For a more extended version with examples, see file “paraly_annotation_guidelines.pdf”.

### 4.6 Model training and evaluation

The dataset for this task could have been treated as either a single-label or multi-label classification problem. In the single-label setup, the labels were simplified into three categories: ``f'' (figurative), ``c'' (concrete), and ``cf'' (a combination of both). However, we opted for the multi-label approach to retain the granularity of the data and better capture instances where multiple labels might apply simultaneously.

To implement the multi-label classification, we used the Flair framework with the CamemBERT large model for document embeddings, see the script train_fc.py. Each sample in the dataset was associated with one or more labels, and a threshold of 0.4 was set for label prediction.

The model was trained for 10 epochs with an initial learning rate of 8e-3, later halved to 4e-3. The training process showed steady improvements, with the training loss decreasing from 1.3293 in the first epoch to 0.0574 in the final epoch. The development F1-score reached a peak of 0.9349, indicating strong overall performance. The final evaluation results are as follows:

• F-score (micro): 0.9349
• F-score (macro): 0.9352
• Accuracy: 0.8831

Class-specific performance (Table 2) showed similarly high results, with F1-scores of 0.9340 for the ``figurative'' class and 0.9365 for the ``concrete'' class:

**Table 2:** Class-specific evaluation of the baseline paraly-classifier.

	Precision	recall	F1-score	support
“figurative”	0.9340	0.9340	0.9340	106
“concrete”	0.9672	0.9077	0.9365	65
micro avg	0.9461	0.9240	0.9349	171
macro avg	0.9506	0.9208	0.9352	171
weighted avg	0.9466	0.9240	0.9349	171
samples avg	0.9578	0.9448	0.9437	171

**Fig. 1:**
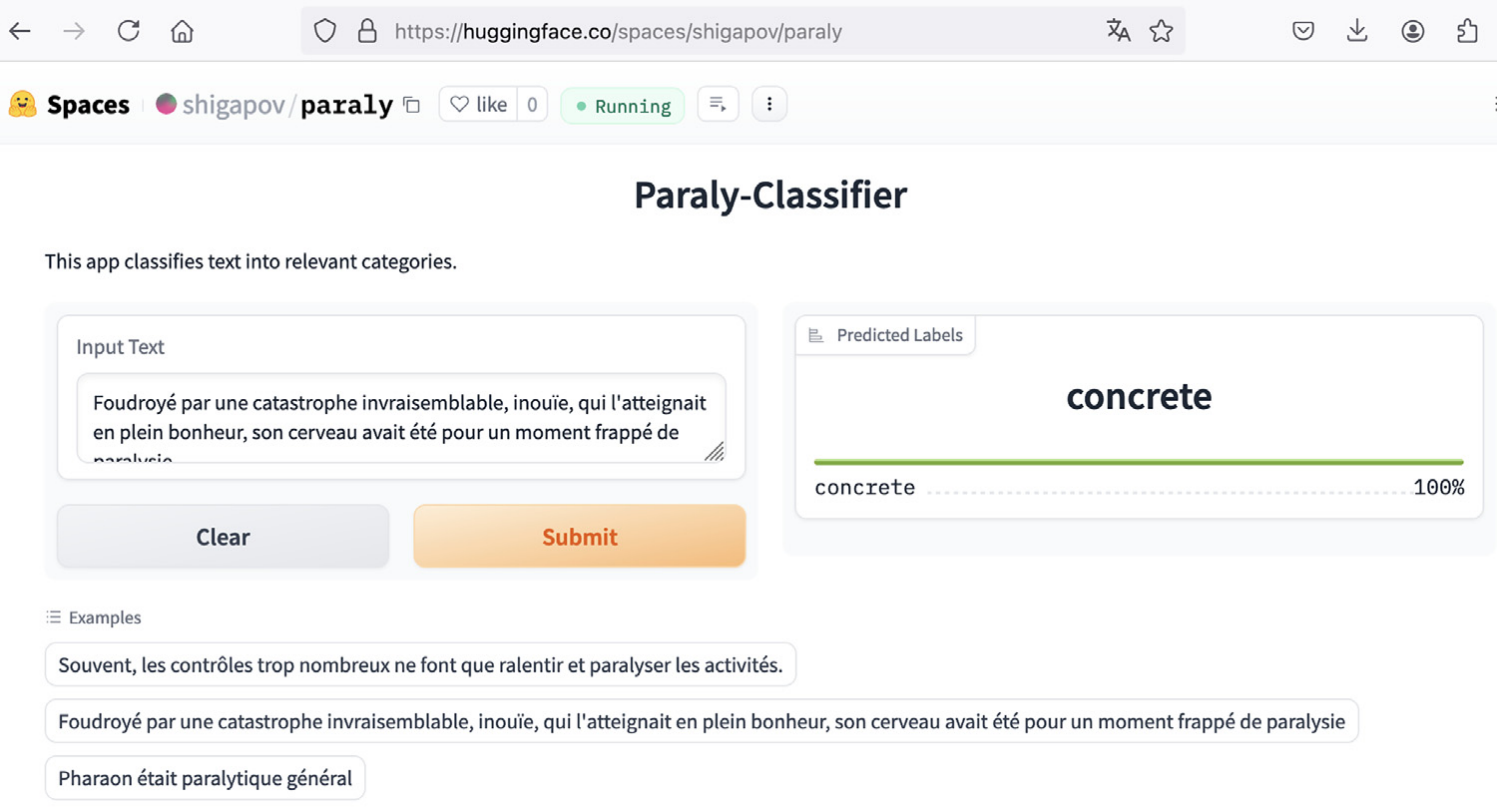
An application with graphical user interface for paraly-classifier.

The trained model is openly published at https://huggingface.co/shigapov/paraly_camembert_large_multilabel under CC BY 4.0 license. We have additionally deployed an application with graphical user interface at Hugging Face Spaces (https://huggingface.co/spaces/shigapov/paraly), see Figure 1.

## Limitations

Due to copyright restrictions and other unresolvable problems, not all texts could be downloaded from the original collection and, hence, were excluded from the project. A list of these errors can be found in the “data/errors” folder.

Furthermore, there are cases in which metadata details are not complete or, as can be seen from individual corrections in the annotation tables, were not properly recorded by the BnF. We did not carry out a systematic revision of the metadata.

The manual annotations were only created by one person (Daniela Kuschel) and were not compared with other annotators.

For various reasons, e.g. the selection of texts in the 19th century by the sdewey classification, not all passages of the overall 1,152 TEI-encoded files have been annotated. Nevertheless, as the annotation tables contain the Archival Resource Key (ARK) the corresponding files can be identified at any time.

## Ethics Statement

We have read and follow the ethical requirements for publication in *Data in Brief* and we confirm that the current work does not involve human subjects, animal experiments, or any data collected from social media platforms.

## CRediT Author Statement

**Daniela Kuschel:** Conceptualization, Methodology, Data Curation, Writing, Original draft preparation. **Renat Shigapov:** Conceptualization, Methodology, Software, Data Curation, Validation, Writing- Reviewing and Editing.

## Data Availability

MADATAParaly: Replication package for exploring the concept of paralysis (fr. ‘paralysie’) in a digital corpus of French Literature (Original data).
